# Anolunula in Fingernails among Patients Infected with HIV

**DOI:** 10.1155/2014/271230

**Published:** 2014-02-23

**Authors:** Pratik Gahalaut, Nitin Mishra, Sandhya Chauhan, Mir Mubashir Ali, Madhur Kant Rastogi, Richa Thakur

**Affiliations:** ^1^Department of Dermatology, SRMSIMS, Bareilly, Uttar Pradesh 243001, India; ^2^Department of Pediatrics, SRMSIMS, Bareilly, Uttar Pradesh 243001, India; ^3^Care Hospital, Hyderabad, India

## Abstract

Lunula is the white, half-moon shaped area seen in proximal ends of some nails. Though a few studies have described the nail changes that can occur in association with HIV infection, none of these paid much attention to lunula. *Aims and Objectives.* To study the lunula in fingernails among HIV infected patients. *Materials and Methods.* An observational, cross-sectional study to record presence of lunula in 168 HIV-positive patients and compare it with age and sex matched 168 healthy HIV-negative control. Anolunula (absence of lunula) in HIV-positive patients was correlated with CD4 counts, stages of HIV infection, time since patient was diagnosed as HIV-positive, and status of antiretroviral therapy. *Results.* Anolunula was present in significantly more fingernails in HIV-positive patients compared to HIV-negative controls. There was a highly significant difference for total anolunula (anolunula in all fingernails) in study and control group. Incidence of total anolunula was directly proportional to the stage of HIV infection, increasing progressively as the HIV infection advances from stage 1 to stage 4. *Conclusion.* Absence of lunula is related to not only HIV infection per se but also the stages of HIV infection.

## 1. Introduction

Lunula is the white, half-moon shaped area seen in proximal ends of some nails [1]. It is the distal part of matrix, which extends beyond the edge of the proximal nail fold [2]. The primary function of the lunula is to define the nail plate's shape [3]. Alterations in morphological features or colour of lunula can be an indication of either a cutaneous or a systemic disorder [4]. Microlunula (diminished size of lunula) and anolunula (absence of lunula) have been described in association with several disorders, listed elsewhere, which include infections like leprosy and HIV [5]. Though a few studies have described the nail changes that can occur in association with HIV infection, none of these paid much attention to lunula [6–8]. Rare case reports of yellow nail syndromes have been cited in the literature for anolunula in HIV infection [9]. Hence, this study was planned to study the association of lunula with HIV infection.

## 2. Aims and Objectives

1. To study the absence of lunula (anolunula) in fingernails among patients infected with HIV,
2. to evaluate the specificity of this finding by comparing it with age and sex matched HIV-negative control subjects,
3. to correlate anolunula (absence of lunula) with different parameters in fingernails of HIV-positive patients.

## 3. Materials and Methods

### 3.1. Design of the Study

This study is an observational, cross-sectional study. Nail changes were recorded after a standardized clinical examination in a predesigned written performa by a single principal investigator.

### 3.2. Setting

The study was done in government district hospital and tertiary care hospital attached to a private medical college.

### 3.3. Subjects

There were 168 patients diagnosed with HIV-1 infection and 168 healthy HIV-negative control subjects having comparable age and sex ratio.

### 3.4. Methodology

A total of 168 patients of HIV presenting at the dermatology department of a government district hospital (Wenlock Government District Hospital, Mangalore, Karnataka state, India) from 2003 to 2005 and a tertiary care hospital attached to a private medical college (SRMSIMS, Bareilly, Uttar Pradesh state, India) from 2006 to 2012 were included in this study based on the below-mentioned exclusion criterion. This was an observational, cross-sectional study with no followup and subjects were examined during either an outpatient visit or admissions. All the patients had proven HIV infection as per guidelines of National AIDS Control Organization of India [10]. This observational study was approved by our institutional ethical committee.

Controls were 168 HIV-negative healthy age and sex matched individuals examined by the same investigator during the study period. Majority of controls were either healthy volunteers or relatives of HIV patients who consulted or demanded HIV testing. They were included as a control on the basis of a negative HIV ELISA test in past 3 months.

All the patients and controls gave oral informed consent for examination and photography of their fingernails. Patients aged <18 years were excluded from the study group. Suspected clinical cases of proximal onychomycosis were confirmed by either a positive KOH nail clipping examination and/or fungal culture. Such positive fingernails were excluded from the study to remove any ambiguity as it was deemed that the change in lunulae for such cases may be due to onychomycosis.

Only the presence or absence of lunula was recorded without any consideration for colour/shape/size of lunulae or any other nail findings. Demographic data, duration of HIV positivity, details of antiretroviral therapy, or any other prophylactic treatment was recorded. We tried to correlate anolunula in HIV-positive patients with CD4 counts, stages of HIV infection, time since patient was diagnosed HIV-positive, and status of antiretroviral therapy in these HIV-positive patients. CD4 counts were available in 150/168 HIV-positive patients. For comparison, HIV-positive patients were divided into 2 groups based on CD4 counts, either ≤350 or >350. HIV infection was classified into Stages 1, 2, 3, and 4 based on WHO classification [11]. Further findings were compared to time since patients were diagnosed HIV-positive. Based on either the laboratory investigations or its absence, the history as told by patient, study population was divided into 2 groups, HIV-positive for ≤6 months or >6 months.

Occupational details were noted for both study and control groups. Special attention was paid to whether occupation resulted in frequent mechanical trauma to the finger nails. The frequency of anolunula was compared between two groups using the Fisher's exact test or Chi square test (whichever appropriate). Results are reported as mean ± S.D. Means were compared using independent sample 2-tailed *t*-test. Statistical analysis was done with the help of Graph Pad prism 6.0 software.

## 4. Results

168 HIV-positive patients included 130 males and 38 females with a mean age of 33.16 ± 6.78 years (range 24 to 45 years). The mean duration of HIV infection was 1.2 ± 0.9 years. (range from 1 month to 5 years). As per WHO classification of HIV infection, 6 patients were in Stage 1, 32 in Stage 2, and 62 and 68 patients were in Stages 3 and 4, respectively.

The HIV-negative control group consisted of 123 males and 45 females with a mean age of 34.08 ± 6.23 years (range 19 to 44 years). This difference in sex between the two groups was not statistically significant (*P* = .4480) and the mean age was comparable in both the groups.

CD4 counts were available for 150 study patients. Out of these, 130 patients had a CD4 count of ≤350. Remaining 20 patients had a CD4 counts of >350. Antiretroviral agents were started in 54 patients. 43 patients were on prophylactic treatment with a combination of trimethoprim and sulphamethoxazole.

100/168 study patients were unemployed. Among 68 employed patient of HIV, working environment of 42 patients involved frequent mechanical/chemical trauma. In control group, 122/168 subjects were employed and only 26 subjects were exposed to frequent mechanical/chemical trauma.

**Figure 1:**
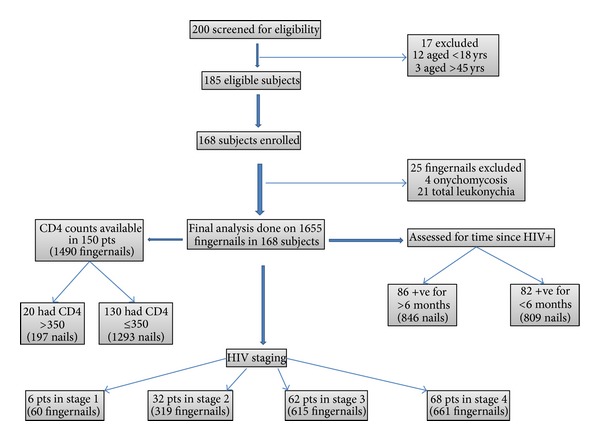
Schematic flow chart of patients in study group.

1655 fingernails from 168 patients were included in final analysis compared to 1676 fingernails from 168 controls.  Figure 1 describes the schematic flow chart of study group. A clinical diagnosis of proximal onychomycosis, obscuring lunula, was confirmed in 4 fingernails of 4 HIV-positive patients. Hence, these fingernails were removed from final analysis. None of the control subjects had proximal onychomycosis. Total leukonychia was observed in 21 fingernails of study patients compared to 4 of control subjects. These were also excluded from the final analysis. Hence, 25 fingernails were excluded from study group and 4 fingernails from control group.

### 4.1. Changes in Lunula

**Figure 2:**
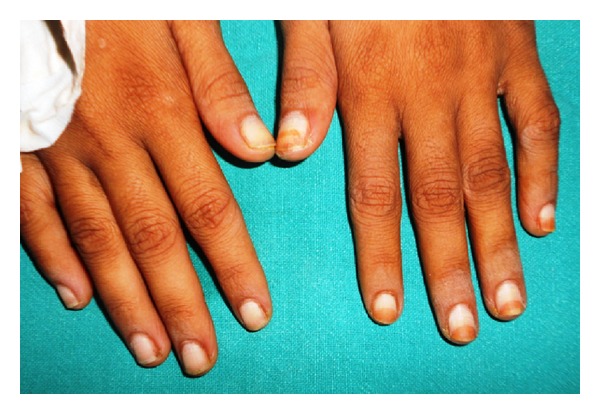
Total anolunula in a HIV-positive patient.

Table 1 gives the description of anolunula in various fingernails in study and control groups. Only 8/168 (4.76%) patients had lunula in all 10 fingers among study patients compared to 77/168 (45.83%) in control group (*P* = .001). In the study group, 76/168 (45.25%) patients had anolunula in all 10 fingers (total anolunula) compared to 6/168 (3.57%) controls (*P* = .0001). 130/168 patients in study group had anolunula in >5 fingernails compared to 32/168 of controls (*P* = .0001). Anolunula was present in 7.714 ± 2.779 mean nails in 168 patients compared to 2.52 ± 2.83 mean nails in 168 controls (*P* = .0001, *t* = 16.939, df = 334).  Figure 2 shows total anolunula in all the 10 fingernails in an HIV-positive patient.

The study population of 168 HIV-positive patients was further evaluated and analyzed to compare association of anolunula with several other factors. Anolunula in all 10 fingernails (total anolunula) was observed in 4/20 (25%) of patients having CD4 count >350 compared to 60/130 (46.15%) patients with CD4 counts ≤350. This difference was statistically significant (*P* = .0305).

In the present study, total anolunula was documented in 45/86 (52.32%) patients having HIV for >6 months compared to 29/82 (35.36%) patients having HIV for <6 months in this study. Significantly more patients had total anolunula if they had HIV for more than 6 months (*P* = .0304).

As per Table 2, presence of anolunula in various fingernails among HIV-positive subjects was very significantly correlated with different stages of HIV (*P* < .0001, Chi = 53.721, df = 3). Incidence of anolunula was directly related to the stage of HIV and it increased proportionately with the severity of stage of HIV infection. However, total anolunula failed to show any statistical significance with stages of HIV.

In the study group, only 54/168 HIV-positive patients were on antiretroviral therapy (ART). Total anolunula was seen in 22/54 patients on ART and in 52/114 patients who were not on ART. Though total anolunula was seen more commonly in patients who were not on ART, there was no significant correlation between ART and total anolunula (*P* = .6190).

## 5. Discussion

Although lunula is often not visible on all finger, and toes, it is most consistently observed on the thumb, the index finger, and the great toe [1, 2, 4]. On the whole it is rarely seen on the toes [2]. The size of lunula is variable not only among persons but also for each digit in the same person [5]. Lunula is most prominent on the thumb, becoming less prominent in a gradient towards the little finger [2]. Lunular size diminishes with age [5]. Recent studies have reported an increasing tendency of presence of lunulae of all fingers with age until 50–59 years old in males and till 40–49 years old group in females [4].

Histologically, lunula has several unique features. Although the granular layer is absent in lunula when compared to the proximal nail fold, lunular epithelium is thicker compared to the adjacent nail bed [5, 12]. Lunula is a keratogenous zone as it contains parakeratotic onychocytes [13]. Diffraction of light from these parakeratotic onychocytes within lunula and diminished visibility of dermal capillaries in lunula because of thicker epithelium may contribute to the white appearance of lunula [5, 12]. Besides, nail plate is thinner over lunula than the adjacent nail bed and less firmly attached over lunula; this allows light to be reflected from the nail plate-lunula interface and may produce the whiteness of lunula [2, 5, 14].

Alterations in morphological features or colour of lunula can be an indication of either a cutaneous or a systemic disorder [4]. Most of these conditions are acquired. Lunula may be lost or become smaller progressively in persons who had lunula of normal size previously [5]. Often the microlunula or anolunula uniformly occurs in all digits [5].

Earlier study hypothesized that onychoschizia, discolouration, and brittle nails reflect various conditions observed in HIV [8]. Absence of lunulae in 3.57% of normal controls in the present study is comparable to the study by Kim et al. in Korean population [4].

In the present study, anolunula was present in significantly more fingernails in HIV-positive patients compared to HIV-negative controls. There was a highly significant difference in the incidence of total anolunula in study and control group. This brings forth a valid argument; whether total anolunula is a novel marker of HIV infection? Further total anolunula increases with the time period in an HIV-positive patient. The result of present study, that incidence of total anolunula increases significantly in patients diagnosed with HIV for >6 months duration when compared to patients diagnosed with HIV for <6 months, lends support to this hypothesis that anolunula may be a novel marker of HIV infection. Moreover, incidence of total anolunula is directly proportional to the stage of HIV infection; increasing as the HIV infection advances from stage 1 to 4. Though the present study failed to report significant association of anolunula with antiretroviral therapy (ART), earlier studies have suggested that long exposure to ART drugs could be a causal factor in lunular or nail dyschromias [8]. Besides, a decreased rate of nail growth has been observed in patients treated with zidovudine [15]. A smaller study sample may be the probable reason for this finding in the present study.

Though it is difficult to ascertain the exact cause of anolunula in HIV infection at present, alterations in vascular or lymphatic systemic may be the most possible explanation. It is well documented that vessels in proximal nail folds may be affected by systemic diseases. Endothelial dysfunction and injury have been described in HIV infection [16, 17]. HIV itself has been implicated in premature atherosclerosis observed in young adults in the absence of major risk factors or treatment with protease inhibitors [18]. Atherogenesis may be stimulated by HIV-infected monocyte-macrophages possibly via altered leukocyte adhesion or arteritis [19]. The vascular or endothelial injury may affect nail growth and subsequently result in anolunula. But this can only be proven by observing histopathological changes occurring in nails in HIV-positive patients.

Functional lymphatic impairments may be another etiologic explanation for anolunula akin to yellow nail syndrome (YNS). In YNS, various changes produce an overall rate of nail growth that is usually less than 1/10th of the normal [20, 21]. Disappearance of lunulae may occur because of the slow growth of the nail [21]. Stage specific transcriptional signatures have been detected in lymphatics during HIV-1 expression [22].

The present study shows a highly significant association of anolunula in a study population of HIV infected patients compared to normal controls. Nevertheless, our study cannot confirm that the findings are specifically due to HIV infection. A comparison with patients having other chronic illnesses could help to distinguish if this finding is specifically associated with HIV infection. No attempt was made to diagnose and exclude YNS in the present study. Yellow nail syndrome (YNS) is a rare disorder of unknown cause characterized by the triad of yellow discoloration and thickened nails with slow growth, chronic lymphedema, and respiratory manifestations, such as bronchiectasis or pleural effusion [20, 21]. Affected nails in YNS frequently show ridging owing to interrupted growth and onycholysis can occur in one or more nails [21]. It is noteworthy that none of the fingernails in the present study was thickened or had onycholysis. However no other systemic illness or disorder was excluded or searched among the study subjects in the present study.

We did not consider the individual size of fingernails in our study. Lunula is more frequently visible in longer nails. Amer et al. suggested that a large sized nail must be formed from a large matrix that leads to forward extension of the distal matrix as visible lunula [23]. On the other hand a small sized nail has an invisible lunula [23]. Racial variations in the normal size of the lunula have been mentioned in the past and incidentally macrolunula (enlarged lunula) has been observed as a normal variant in persons from India [5].

Various opportunistic infections occur in HIV. Their incidence increases with advancing stages of HIV infection. The presence of anolunula in present study may be due to some of these opportunistic infections. Due to logical reasons we were not able to exclude these confounding biases. But still, these few limitations cannot negate the results of our present study. However a larger, may be multicentric, study is needed to support our findings.

## 6. Conclusion

A visible lunula is a good sign as it may reflect matrix size in healthy subjects [23]. To conclude, nail examination of HIV infected patients is necessary and valuable for a treating physician. Absence of lunula is related to not only HIV infection per se but also to the stage of HIV infection. Further studies are warranted to find out the exact cause of anolunula in HIV infection.

## Figures and Tables

**Table 1:** Anolunula in 168 study and control subjects.

Fingernail	Cases (%)	Controls (%)	*P* value
Right thumb	98 (58.33)	4 (2.38)	.0001
Right index	108 (64.28)	18 (10.71)	.0001
Right middle	132 (78.57)	28 (16.66)	.0001
Right ring	146 (86.90)	68 (40.47)	.0001
Right little	156 (92.85)	82 (48.80)	.0001
Left thumb	108 (64.28)	8 (4.76)	.0001
Left index	118 (70.24)	26 (15.47)	.0001
Left middle	130 (77.38)	34 (20.24)	.0001
Left ring	150 (89.28)	72 (43.63)	.0001
Left little	152 (90.47)	87 (54.37)	.0001

Total (all fingernails)	76 (45.24)	6 (3.57)	.0001

**Table 2:** Description of anolunula in different fingernails for different stages of HIV.

Stage of HIV	Anolunula present (%)	Anolunula absent (%)	Total fingernails
Stage 1	20 (33.33)	40 (66.67)	60
Stage 2	130 (40.75)	189 (59.25)	319
Stage 3	300 (48.78)	315 (51.21)	615
Stage 4	410 (62.02)	251 (37.98)	661

Total	860 (51.96)	795 (48.04)	1655

*P* < .0001; Chi = 53.721; df = 3.
